# Quantitative Assessment of Myocardial Perfusion in Physiological and Pathological Hypertrophy Using Myocardial Contrast Echocardiography

**DOI:** 10.1002/clc.70285

**Published:** 2026-04-30

**Authors:** Liu Chunyao, Jiang Ruihan, Li Kai, Lin Mingming, Wang Xiaofan, Lv Qifeng, Song Xiaoxia, Tian Yu, Sun Juanjuan, Sun Pin

**Affiliations:** ^1^ Department of Cardiac Ultrasound The Affiliated Hospital of Qingdao University Qingdao China

**Keywords:** myocardial contrast echocardiography, myocardial microcirculation, pathological hypertrophy, physiological hypertrophy

## Abstract

**Objective:**

To quantitatively evaluate myocardial perfusion levels in patients with physiological and pathological myocardial hypertrophy using myocardial contrast echocardiography (MCE), and to investigate the diagnostic value of MCE parameters for differentiating between these conditions.

**Methods:**

From June 2023 to December 2024, 25 hypertensive patients with myocardial hypertrophy (pathological hypertrophy group), 25 healthy athletes (physiological hypertrophy group), and 25 healthy controls were enrolled. All participants underwent two‐dimensional echocardiography and MCE. Myocardial perfusion parameters—peak intensity (*A*‐value), wash‐in slope (*β*‐value), and myocardial blood flow (MBF)—were quantified using a 17‐segment model. Differences in perfusion parameters were compared, and receiver operating characteristic (ROC) curve analysis was performed to evaluate diagnostic efficacy.

**Results:**

Compared with controls, the physiological hypertrophy group showed significantly increased peak intensity (*A*‐value) and MBF (*p* < 0.05), whereas the pathological hypertrophy group exhibited decreased A‐value, slope rate *(β*‐value), and MBF (*p* < 0.05). Intragroup segmental analysis revealed that in pathological hypertrophy, the basal segments had significantly lower A‐value and MBF compared to mid and apical segments (*p* < 0.05). Intermural comparison demonstrated that in both control and physiological hypertrophy groups, the free wall had lower *A*‐value and MBF than the septum (*p* < 0.05). In contrast, pathological hypertrophy showed reduced *A*‐value but increased *β*‐value in the free wall (*p* < 0.05). ROC curve analysis identified an optimal cutoff value of *A* > 8.13 dB (AUC = 0.904) for discriminating exercise‐induced physiological hypertrophy from hypertension‐induced pathological hypertrophy.

**Conclusion:**

MCE enables quantitative assessment of myocardial perfusion in hypertrophic patients. The *A* value, reflecting microvascular density, serves as a reliable discriminator between physiological and pathological hypertrophy.

## Graphical abstract

Myocardial hypertrophy can be classified into physiological and pathological types based on etiology, with significant differences in clinical outcomes. This study utilized myocardial contrast echocardiography to quantitatively assess myocardial microcirculatory perfusion in patients with different types of hypertrophy. The results demonstrate that myocardial contrast echocardiography perfusion parameters—particularly the A value, which reflects microvascular density—can effectively differentiate between physiological and pathological myocardial hypertrophy, providing an important reference for early clinical diagnosis and differentiation.

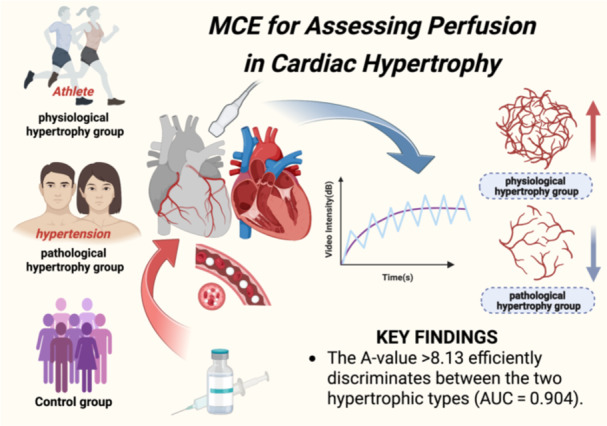

## 1 Introduction

Myocardial hypertrophy refers to a series of pathophysiological changes, including cardiomyocyte thickening and enhanced contractility, that occur in response to chronic excessive workload to maintain normal circulatory blood volume [1]. Based on etiology, it can be classified into physiological myocardial hypertrophy and pathological myocardial hypertrophy. Physiological myocardial hypertrophy is a reversible adaptive response, commonly observed in athletes engaged in long‐term physical training. In contrast, pathological myocardial hypertrophy arises from diverse causes, including pressure overload (e.g., hypertension), volume overload (e.g., valvular regurgitation), specific cardiomyopathies, and compensatory hypertrophy secondary to myocardial hypoxia. This pathological form is often accompanied by myocardial fibrosis and represents an irreversible thickening [2]. Early studies have demonstrated that physiological hypertrophy exhibits proportional microvascular angiogenesis matching the degree of myocardial thickening, whereas pathological hypertrophy shows disproportionate vascular growth, resulting in impaired myocardial perfusion and subsequent cardiac dysfunction [3]. Accumulating evidence indicates that myocardial hypertrophy is an independent risk factor for increased cardiovascular morbidity and mortality.

Myocardial contrast echocardiography (MCE) is a noninvasive technique for evaluating coronary microcirculatory perfusion. It involves peripheral venous injection of ultrasound contrast agents, with qualitative assessment of myocardial microvascular perfusion based on contrast intensity. With technological advancements, quantitative evaluation of microcirculation has become possible by analyzing the intensity‐time relationship of contrast agents to calculate myocardial blood flow [4, 5].

This study employs MCE to assess myocardial perfusion in patients with cardiac hypertrophy, aiming to characterize microcirculatory patterns across different hypertrophy subtypes. We hypothesize that pathological myocardial hypertrophy is associated with impaired microcirculatory perfusion, whereas physiological hypertrophy maintains normal myocardial microvascular perfusion.

## 2 Materials and Methods

### 2.1 Study Population

We enrolled 25 hypertensive patients who underwent myocardial contrast echocardiography (MCE) at the Affiliated Hospital of Qingdao University between June 2023 and December 2024, designated as the pathological hypertrophy group (17 males, 8 females; mean age 61.12 ± 12.64 years). Inclusion criteria: ① Systolic blood pressure (SBP) ≥ 140 mmHg and/or diastolic blood pressure (DBP) ≥ 90 mmHg upon examination, or a documented history of hypertension requiring current antihypertensive pharmacological treatment; ② Diagnosis of left ventricular hypertrophy (LVH) based on left ventricular mass index (LVMI) according to Chinese guidelines [6, 7] (LVMI ≥ 109 g/m² in males, ≥ 105 g/m² in females); ③ Exclusion of myocardial hypertrophy due to other causes, coronary artery disease (CAD), ischemic cardiomyopathy, myocardial infarction, or other cardiac conditions affecting myocardial perfusion; ④ Good image quality.

Additionally, 25 elite athletes were included as the physiological hypertrophy group (15 males, 10 females; mean age 54.92 ± 11.80 years). Inclusion criteria: ① Long‐distance runners with a training history of ≥8 years and ≥3 h of daily exercise; ② Meeting LVH diagnostic criteria per Chinese guidelines [6, 7] (LVMI ≥ 109 g/m² in males, ≥105 g/m² in females); ③ No significant abnormalities detected on electrocardiogram (ECG) examination; ④ Exclusion of primary hypertension, hypertrophic cardiomyopathy, CAD, ischemic cardiomyopathy, myocardial infarction, or other perfusion‐affecting cardiac diseases; ⑤ Good image quality.

A control group of 25 healthy individuals was selected, matched for sex and age with both hypertrophy groups. Inclusion criteria for the control group required participants to be healthy subjects undergoing routine physical examination with no significant abnormalities detected on general echocardiography and ECG examination. Exclusion criteria included primary hypertension, hypertrophic cardiomyopathy, and other cardiac conditions affecting myocardial perfusion, such as coronary atherosclerotic heart disease, ischemic cardiomyopathy, and myocardial infarction.

To systematically rule out hypertrophic cardiomyopathy (HCM), a standardized diagnostic protocol was applied to all participants in both the athletic cohort and the healthy control group:

(1) Clinical screening: Each participant underwent comprehensive clinical evaluation including detailed personal and familial cardiovascular history, documentation of syncopal events, and assessment of exercise‑associated symptoms. Standard 12‑lead resting electrocardiography was performed in all cases.

(2) Comprehensive echocardiographic assessment: Following the American Society of Echocardiography guidelines, complete echocardiographic measurements of left ventricular wall thickness were obtained. Particular attention was directed toward detecting asymmetric septal hypertrophy (septal‑to‑posterior wall thickness ratio ≥ 1.3), systolic anterior motion of the mitral valve (SAM), and left ventricular outflow tract obstruction—all established hallmarks of HCM. Participants exhibiting echocardiographic findings suggestive of HCM were excluded from the study and referred for advanced cardiac magnetic resonance imaging (CMR) and genetic counseling.

Ultimately, none of the enrolled athletes or control subjects demonstrated echocardiographic features satisfying the diagnostic criteria for HCM as defined by the 2020 AHA/ACC guidelines [8].

All participants provided written informed consent, and the study protocol was approved by the Ethics Committee of the Affiliated Hospital of Qingdao University (Approval No.: QYFYEC2025‐28).

### 2.2 Equipment

All examinations were performed using a Philips EPIQ 7C ultrasound system (Philips Medical Systems, Andover, MA) equipped with an X5‐1 transducer (frequency range 1–5 MHz) featuring contrast‐specific imaging mode for myocardial perfusion assessment. All images were acquired and subsequently analyzed offline using Philips QLAB 13.0 software (Philips Healthcare).

All study participants were examined in the left lateral decubitus position while maintaining spontaneous respiration. Following establishment of intravenous access via the right antecubital vein, continuous electrocardiographic monitoring was implemented throughout the examination procedure.

## 3 Routine Echocardiographic Examination

Following acquisition of optimal 2D ultrasound images from three consecutive cardiac cycles, the following parameters were obtained: left ventricular end diastolic diameter (LVEDD), left ventricular end systolic diameter (LVESD), interventricular septum thickness at end diastole (IVSd), and left ventricular posterior wall thickness at end diastole (LVPWTd) were measured at the parasternal long‐axis view.

At the apical 4‐chamber view under pulsed‐wave Doppler mode, the peak early diastolic (E‐wave) and late diastolic (A‐wave) mitral inflow velocities were recorded, along with the septal mitral annular early diastolic velocity (e'). The E/A ratio and E/e' ratio were subsequently calculated.

Left ventricular ejection fraction (LVEF) was determined using the modified Simpson's biplane method. Left ventricular mass index (LVMI) was calculated according to the following formula:

LVMI = (0.8 × 1.04 × [(IVSd + LVEDD + LVPWTd)³ − LVEDD³] + 0.6)/(height⁰·⁷²⁵ × weight⁰·⁴²⁵ × 0.007184). Relative wall thickness (RWT) was calculated using the following formula: RWT = (2 × LVPWTd)/LVEDD. Based on myocardial geometry criteria established for the Chinese population [7], the presence of LVH was further classified: concentric hypertrophy (CH) was defined as LVH with an RWT > 0.51, and eccentric hypertrophy (EH) was defined as LVH with an RWT ≤ 0.51. LVH was diagnosed based on LVMI ≥ 109 g/m² in males, ≥ 105 g/m²in females.

## 4 MCE Examination and Image Analysis Protocol

For MCE, the contrast agent was prepared by adding 5 mL of SonoVue (Bracco Imaging) to normal saline, followed by vigorous agitation for 30 s to form a stable microbubble suspension. During the procedure, 1 mL of the prepared contrast agent was slowly administered via a peripheral vein (preferentially the right antecubital vein), followed by a 5 mL normal saline flush. Left ventricular myocardial opacification was monitored in real‐time. Upon achieving stable contrast enhancement, high mechanical index “flash” imaging was manually triggered to destroy microbubbles. Dynamic images were acquired for three cardiac cycles before flash and ten cardiac cycles after flash in apical four‐chamber, three‐chamber, and two‐chamber views.

**Figure 1:**
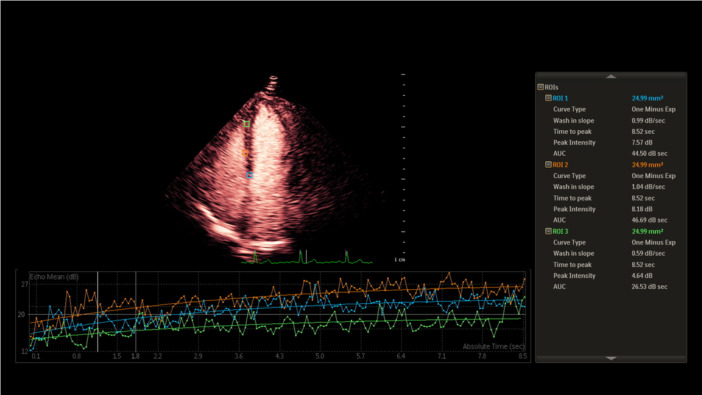
Quantitative Myocardial Contrast Echocardiography (MCE) Analysis in Apical Four‐Chamber View. AUC, area under the curve; ROI, regions of interest.

Offline analysis was performed using QLAB 13.0 software (Philips Medical Systems). The left ventricular myocardium was divided into segments according to the 17‐segment model, with regions of interest (ROIs) placed at the mid‐myocardial layer of each segment. Motion compensation algorithms and frame‐by‐frame adjustments were applied to ensure accurate ROI positioning throughout the cardiac cycle. Time‐intensity curves were generated and fitted to the single‐exponential function: y(t) = A×(1‐e^(−βt)) + C, where *A* represents peak intensity (reflecting microvascular blood volume), *β* denotes wash‐in slope (representing microvascular flux rate), and myocardial blood flow (MBF) was calculated as A × β (Figure 1). All parameters were measured in triplicate and averaged to ensure reproducibility.

### 4.1 Statistical Analysis

Statistical analysis was performed using SPSS 26.0 software. Categorical data are expressed as numbers and percentages [*n* (%)], and group comparisons were conducted using the chi‐square test. Normally distributed continuous variables were expressed as mean ± standard deviation (SD) (X ± S) and compared using one‐way ANOVA, while non‐normally distributed continuous variables were expressed as median (*P*_25_, *P*_75_) and compared using Kruskal–Wallis *H* test. Receiver operating characteristic (ROC) curve analysis was employed to determine optimal cutoff values for differentiating various types of myocardial hypertrophy. The reproducibility of MCE perfusion parameters was assessed using intraclass correlation coefficient (ICC). A *p*‐value < 0.05 was considered statistically significant.

## 5 Results

### 5.1 Baseline Characteristics and Conventional Echocardiographic Parameters

This study enrolled 75 participants categorized into: pathological hypertrophy (*n* = 25, 61.12 ± 12.64 years), physiological hypertrophy (*n* = 25, 54.92 ± 11.80 years), and controls (*n* = 25, 54.44 ± 14.50 years). The pathological hypertrophy group showed significantly higher systolic blood pressure versus both controls (*p* < 0.05) and physiological hypertrophy (*p* < 0.05), along with elevated heart rate compared to controls (*p* < 0.05). No significant intergroup differences existed in other parameters or baseline characteristics (all *p* > 0.05, Table 1), confirming appropriate cohort matching.

**Table 1:** Baseline characteristics of the groups.

Characteristics	Physiological hypertrophy group (*n* = 25)	Pathological hypertrophy group (*n* = 25)	Control group (*n* = 25)	*p*
Age (years)	54.92 ± 11.80	61.12 ± 12.64	54.44 ± 14.50	0.137
BMI (kg/m^2^)	25.33 ± 3.35	24.95 ± 2.74	26.76 ± 2.64	0.077
SBP (mmHg)	127.60 ± 15.64	136.36 ± 15.54* ^,^ #	121.56 ± 12.93	0.002
DBP (mmHg)	77.32 ± 9.85	79.96 ± 10.47	76.08 ± 11.17	0.416
HR (bpm)	75.24 ± 12.66	78.20 ± 10.28*	70.28 ± 6.63	0.025
LVEF (%)	64.25 ± 3.21	64.36 ± 3.48	66.12 ± 3.12	0.083
e'(cm/s)	8.63 ± 0.99	6.22 ± 1.86* ^,^ #	9.44 ± 1.15	0.001
E/e'	10.42 ± 2.65	14.06 ± 3.78* ^,^ #	9.00 ± 2.52	0.001
E/A	0.99 ± 0.18	0.71 ± 0.25* ^,^ #	1.00 ± 0.16	0.001
IVSd(cm)	1.29 ± 0.06*	1.36 ± 1.01* ^,^ #	1.07 ± 0.06	0.001
LVPWTd(cm)	1.14 ± 0.05*	1.24 ± 0.06* ^,^ #	0.98 ± 0.07	0.001
RWT	0.48 ± 0.03*	0.54 ± 0.05* ^,^ #	0.44 ± 0.04	0.001

*Note:* Data are mean ± standard deviation.

Abbreviations: BMI, body mass index; DBP, diastolic blood pressure; HR, heart rate; IVSd, interventricular septum thickness at end diastole; LVEF, left ventricular ejection fraction; LVPWTd, left ventricular posterior wall thickness at end diastole; SBP, systolic blood pressure.

* compared to control group, *p* < 0.05.

^#^ compared to physiological hypertrophy group, *p* < 0.05.

In contrast, the pathological hypertrophy group demonstrated significantly reduced e′, E/e′, and E/A ratios versus both controls and the physiological hypertrophy group (all *p* < 0.05). The pathological hypertrophy group showed significantly greater IVSd, LVPWTd, and RWT compared to both the control group and the physiological hypertrophy group (all *p* < 0.05). Meanwhile, the physiological hypertrophy group also exhibited significantly higher IVSd, LVPWTd, and RWT than the control group (all *p* < 0.05). No other parameters differed significantly among groups (all *p* > 0.05, Table 1).

**Figure 2:**
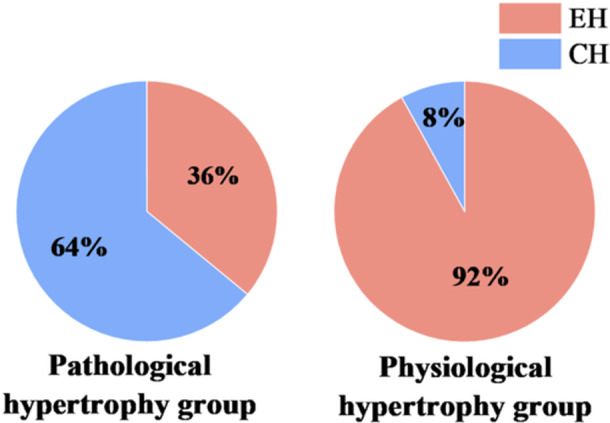
Comparison of left ventricular geometric patterns between physiological and pathological hypertrophy groups. EH, eccentric hypertrophy; CH, concentric hypertrophy.

Additionally, in the pathological hypertrophy group, 16 cases (64%) exhibited CH and nine cases (36%) exhibited EH. Conversely, in the physiological hypertrophy group, two cases (8%) were classified as CH and 23 cases (92%) as EH. Chi‐square test indicated a statistically significant difference in left ventricular geometric patterns between the two groups (χ² = 12.83, *p* < 0.001, Figure 2).

### 5.2 MCE Parameters

The left ventricular myocardium was segmented using the 17‐segment model, with poorly visualized segments excluded, yielding a total of 1035 analyzable segments (control group: 358; physiological hypertrophy group: 341; pathological hypertrophy group: 336).

### 5.3 Global Perfusion

**Figure 3:**
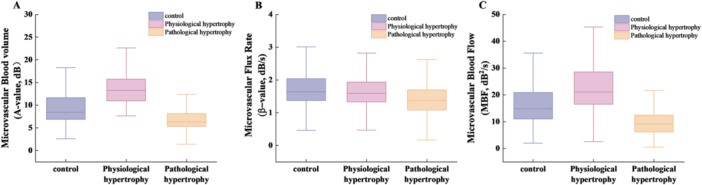
Quantitative MCE perfusion data of the left ventricular myocardium in the control group, physiological myocardial hypertrophy group, and pathological myocardial hypertrophy group. Data include: (A) microvascular blood volume, (B) microvascular flux rate, and (C) microvascular blood flow.

In comparisons of global left ventricular myocardial microcirculatory perfusion parameters, the physiological hypertrophy group exhibited significantly higher *A* value and MBF than the control group (*p* < 0.001), whereas the pathological hypertrophy group showed significantly lower *A* value, *β* value, and MBF (*p* < 0.001). No other parameters differed significantly between groups. Moreover, compared with the physiological hypertrophy group, the pathological hypertrophy group demonstrated significantly reduced *A* value, *β* value, and MBF (*p* < 0.001) (Table 2, Figure 3).

**Table 2:** Comparison of MCE parameters in three groups (*n* = 25).

Parameters	Physiological hypertrophy group	Pathological hypertrophy group	Control group	*p*
***A*‐value (dB)**				
Overall segments	12.96 (10.16, 16.29)*	6.22 (4.90, 8.30)* ^,^ ^#^	8.46 (6.75, 11.53)	*p* < 0.001
Basal segments	13.13 (10.57, 16.05)*	5.97 (4.52, 7.73)* ^,^ #	8.50 (6.78, 11.83)	*p* < 0.001
Middle segments	12.77 (9.69, 15.84)*	6.46 (5.09, 8.86)* ^,^ # ^,^ ^b^	8.51 (6.79, 11.33)	*p* < 0.001
Apical segments	12.92 (10.23, 16.91)*	6.31 (5.16, 8.12)* ^,^ # ^,^ ^b^	8.33 (6.63, 11.21)	*p* < 0.001
IVS	14.09 (10.89, 17.94)*	6.45 (5.31, 9.05)* ^,^ ^#^	9.05 (7.20, 12.28)	*p* < 0.001
LVFW	12.67 (10.02, 15.44)* ^,^ ^I^	6.11 (4.69, 8.01)* ^,^ ^#^ ^,^ ^I^	6.46 (5.09, 8.86)^I^	*p* < 0.001
***β*‐value (dB/s)**				
Overall segments	1.56 (1.23, 1.95)	1.35 (0.96, 1.73)* ^,^ #	1.64 (1.23, 2.13)	*p* < 0.001
Basal segments	1.54 (1.24, 1.96)	1.35 (0.93, 1.75)* ^,^ #	1.62 (1.22, 2.15)	*p* < 0.001
Middle segments	1.55 (1.23, 1.93)	1.35 (0.98, 1.76)* ^,^ #	1.63 (1.21, 2.18)	*p* < 0.001
Apical segments	1.57 (1.23, 1.96)	1.32 (1.01, 1.71)* ^,^ #	1.67 (1.25, 2.08)	*p* < 0.001
IVS	1.56 (1.27, 1.90)	1.23 (0.93, 1.64)* ^,^ #	1.62 (1.28, 2.03)	*p* < 0.001
LVFW	1.56 (1.21, 1.98)	1.39 (0.99, 1.78)* ^,^ # ^,^ I	1.65 (1.21, 2.18)	*p* < 0.001
**MBF (dB** ^**2**^ **/s)**				
Overall segments	20.59 (14.82, 29.03)*	8.75 (5.32, 12.70)* ^,^ #	14.75 (9.91, 21.24)	*p* < 0.001
Basal segments	21.15 (15.49, 30.70)*	8.13 (4.99, 12.43)* ^,^ #	14.55 (9.81, 21.82)	*p* < 0.001
Middle segments	20.49 (13.56, 28.39)*	8.94 (5.56, 13.04)* ^,^ #	14.74 (10.09, 21.51)	*p* < 0.001
Apical segments	20.14 (15.11, 27.60)*	8.87 (5.38, 12.58)* ^,^ #	15.28 (9.92, 20.50)	*p* < 0.001
IVS	22.56 (16.15, 31.72)*	8.76 (5.29, 12.61)* ^,^ #	15.97 (10.73, 22.27)	*p* < 0.001
LVFW	19.62 (14.30, 27.72)* ^,^ ^I^	8.73 (5.33, 12.80)* ^,^ #	14.43 (9.31, 20.38)^I^	*p* < 0.001

*Note:* Data are median (*P*_25_, *P*_75_).

Abbreviations: *A*‐value, peak intensity; *β*‐value, wash‐in slope; IVS, interventricular septum; LVFW, left ventricular free wall; MBF, myocardial blood flow.

* compared to control group, *p* < 0.05.

^#^ compared to physiological hypertrophy group, *p* < 0.05.

^b^ compared to basal segments, *p* < 0.05.

^I^ compared to IVS, *p* < 0.05.

### 5.4 Segmental Microcirculatory Perfusion

When comparing perfusion parameters among the basal, mid, and apical segments within each group, only the pathological hypertrophy group exhibited a significantly lower *A* value in the basal segment compared to the mid and apical segments (*p* < 0.05). No other significant differences were observed among segments in the remaining groups (*p* > 0.05).

**Figure 4:**
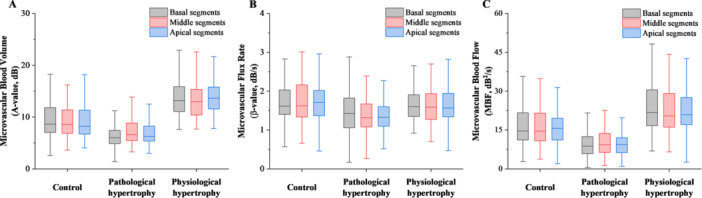
Quantitative MCE perfusion data of the basal, mid, and apical segments of the left ventricular myocardium in the control group, physiological myocardial hypertrophy group, and pathological myocardial hypertrophy group. Data include: (A) microvascular blood volume, (B) microvascular flux rate, and (C) microvascular blood flow.

In intergroup comparisons, relative to the control group, the physiological hypertrophy group showed significantly higher *A* values and MBF across all three segments (*p* < 0.05), whereas the pathological hypertrophy group demonstrated significantly lower *A* value, *β* value, and MBF (*p* < 0.05). Furthermore, compared with the physiological hypertrophy group, the pathological hypertrophy group had significantly reduced *A* value, *β* value, and MBF in all segments (*p* < 0.05), which was consistent with the global MCE perfusion parameters trend observed among groups. No other significant differences were found between groups for the remaining parameters (Table 2, Figure 4).

### 5.5 IVS Versus LVFW Microcirculatory Perfusion

Significant intramural differences were observed between interventricular septum (IVS) and left ventricular free wall (LVFW): controls and physiological hypertrophy groups showed lower LVFW A values and MBF (*p* < 0.05 vs IVS), while pathological hypertrophy demonstrated reduced A values but elevated β values in LVFW (*p* < 0.05).

**Figure 5:**
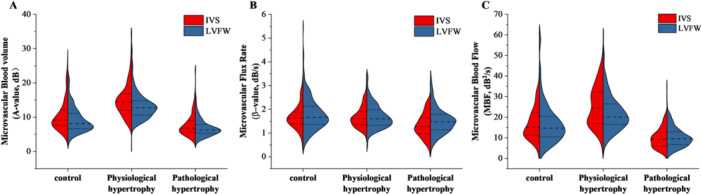
Quantitative MCE perfusion data of the interventricular septum (IVS) and left ventricular free wall (LVFW) in the control group, physiological myocardial hypertrophy group, and pathological myocardial hypertrophy group. Data include: (A) microvascular blood volume, (B) microvascular flux rate, and (C) microvascular blood flow. IVS, interventricular septum; LVFW, left ventricular free wall.

Intergroup comparisons revealed: (1) physiological hypertrophy exhibited elevated A values and MBF in both IVS and LVFW versus controls (*p* < 0.05); (2) pathological hypertrophy displayed reduced A, β, and MBF in both regions (*p* < 0.05); and (3) these findings were consistent with overall MCE perfusion trends. No other parameters showed significant intergroup differences (Table 2, Figure 5).

### 5.6 ROC Curve Analysis

**Figure 6:**
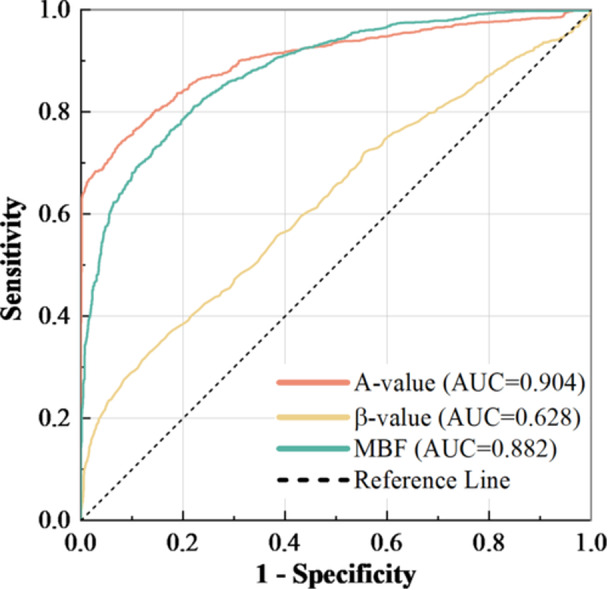
Receiver operating characteristic (ROC) curves demonstrating the diagnostic performance of myocardial contrast echocardiography (MCE) perfusion parameters in differentiating physiological versus pathological myocardial hypertrophy. MBF, myocardial blood flow.

To better differentiate between physiological and pathological myocardial hypertrophy, receiver operating characteristic (ROC) curve analysis was performed for three perfusion parameters. The area under the curve (AUC) values for *A*, *β*, and MBF were 0.904, 0.628, and 0.882, respectively. Among these, parameter A demonstrated superior diagnostic performance. In patients with myocardial hypertrophy, an *A* value > 8.13 indicated physiological hypertrophy with a sensitivity of 0.927 and specificity of 0.735. The ROC curve results are presented in Figure 6.

### 5.7 Reproducibility Analysis

The measured parameters (A, β, and MBF) demonstrated excellent reproducibility, with intra‐observer intraclass correlation coefficients (ICCs) of 0.964, 0.960, and 0.978, respectively, and inter‐observer ICCs of 0.982, 0.982, and 0.987, respectively.

## 6 Discussion

This study aimed to investigate myocardial microcirculatory characteristics in different types of cardiac hypertrophy using myocardial contrast echocardiography (MCE). Early studies have demonstrated that time‐intensity curves derived from contrast perfusion can provide peak intensity (*A*‐value), reflecting myocardial contrast agent concentration and microvascular density (an approximation of myocardial blood volume), and the upslope slope (*β*‐value), representing microcirculatory flow velocity. The product of these parameters yields myocardial blood flow (MBF) [9]. MCE has shown good agreement with positron emission tomography (PET) for microcirculatory assessment while offering advantages of radiation‐free operation, technical simplicity, and high safety [10]. Previous international studies on cardiac hypertrophy primarily focused on hypertrophic segments or major coronary artery territories without accounting for segmental hypertrophy effects [11, 12, 13]. In contrast, our study adopted the 17‐segment model to analyze each cardiac segment systematically, enabling both comprehensive evaluation of global left ventricular perfusion and localized assessment of segmental and regional myocardial perfusion characteristics.

In this study, the physiological hypertrophy group was composed of long‐distance runners. Long‐distance running is classified as a typical endurance exercise, which primarily functions by imposing a volume load on the heart. This load induces longitudinal elongation of cardiomyocytes through a series mechanism, thereby enhancing myocardial contractility and ultimately leading to EH, characterized by ventricular dilation accompanied by moderate wall thickening. Eccentric hypertrophy serves as a physiological adaptive mechanism that increases stroke volume to meet the hemodynamic demands of prolonged high‐intensity exercise [14]. The findings of this study are highly consistent with existing literature: within the physiological hypertrophy group, the proportion of EH was as high as 92%. This discovery strongly corroborates the typical impact of endurance exercise on cardiac remodeling patterns—specifically, a physiological adaptive process predominantly characterized by ventricular dilation [15].

Regarding the left ventricular geometric patterns in the pathological myocardial hypertrophy group, previous studies have established that CH is the typical pattern of myocardial hypertrophy induced by hypertension [16]. In this study, only patients meeting the criteria for LVH were included; therefore, the pathological hypertrophy group primarily exhibited two geometric patterns: CH and EH. Among these, CH accounted for a higher proportion (approximately 64%), which is generally consistent with existing research. However, it is noteworthy that 36% of patients in this group still presented with EH. This phenomenon can mainly be attributed to the lack of standardized pharmacological treatment in some of the included patients. Prolonged and uncontrolled hypertension leads to pressure overload, which activates neurohormonal regulatory mechanisms and induces myocardial fibrosis [16]. This not only impairs diastolic function but can also result in ventricular dilation, manifesting as maladaptive EH. Furthermore, long‐term hypertension may also increase volume load through various mechanisms, subjecting the heart to combined pressure and volume overload [17]. According to the classic study by Ganau et al. [17], eccentric hypertrophy in hypertensive patients is associated with the combined effects of pressure and volume overload, and this geometric pattern typically indicates a poorer clinical prognosis.

In the present study, patients with pathological hypertrophy demonstrated myocardial microcirculatory perfusion defects while maintaining normal‐range ejection fractions comparable to controls. This phenomenon can be attributed to two mechanisms: the ischemic microcirculatory changes predominantly affected the subendocardial myocardium with heterogeneous distribution, sparing epicardial vasculature [18]; and despite myocardial fibrosis impairing contractile function, compensatory enhancement of left ventricular torsion preserved stroke volume within normal limits [19]. However, these patients exhibited significantly impaired diastolic function, resulting from: hypertension‐induced myocardial fibrosis causing structural disorganization and reduced myocardial compliance [19]; and hemodynamic alterations leading to abnormal energy metabolism and impaired ATP production, both contributing to diastolic dysfunction [20].

In this study, the physiological hypertrophy group exhibited significantly higher global left ventricular myocardial *A* values and MBF compared to the control group, whereas the pathological hypertrophy group showed significantly lower *A* values, *β* values, and MBF. These findings suggest that exercise‐induced physiological hypertrophy enhances myocardial microcirculatory blood flow by increasing capillary density to meet metabolic demands, whereas the reduced myocardial perfusion in pathological hypertrophy is associated with both diminished capillary density and altered blood flow velocity. The microcirculatory adaptations in physiological hypertrophy align with the hypothesis proposed by Selthofer et al. [21], indicating that myocardial hypertrophy primarily modulates microcirculatory perfusion through vascular density regulation. Furthermore, earlier work by Di Bello et al. [22] demonstrated that capillary endothelial cell‐related mechanisms in physiological hypertrophy promote angiogenesis, thereby augmenting capillary density. Conversely, the observations in pathological hypertrophy support previous findings that hypertension‐induced pathological hypertrophy involves structural microcirculatory abnormalities—including capillary rarefaction and microvascular remodeling—leading to impaired vasodilation/constriction, increased microvascular resistance, altered flow velocity, and ultimately reduced microcirculatory perfusion [22, 23]. Additionally, hypertension‐driven hemodynamic changes and ventricular wall stress perturbations may disrupt myocardial metabolism, with subsequent metabolite alterations impairing coronary arteriolar network function, further contributing to diminished myocardial blood flow [24, 25].

The intergroup comparison of three‐segment MCE parameters showed consistent trends with the results presented in Table 2. The intragroup comparison among the three segments revealed that in the pathological hypertrophy group, the *A* value of the basal segment was significantly lower than that of the mid and apical segments, while no significant differences were observed in *β* values and MBF among the three segments. These findings suggest that in hypertensive patients, particularly elderly patients with long‐standing hypertension, the basal segment demonstrates significant thickening due to factors such as ventricular wall stress. As previously discussed, this pathological hypertrophy leads to reduced microcirculatory capillary density, primarily resulting from the mismatch between hypertrophied myocardium and capillary angiogenesis during the hypertrophic process [11, 12].

Interventricular septum and free wall MCE parameter comparisons between groups were consistent with the results shown in Table 2. Within‐group comparisons revealed that both healthy controls and physiological hypertrophy patients exhibited superior myocardial capillary density and blood flow in the interventricular septum compared to the free wall. This suggests that physiological hypertrophy does not alter the normal myocardial microcirculatory blood supply mechanism, but rather enhances myocardial blood flow primarily through increased capillary density. In contrast, the pathological hypertrophy group (hypertension‐induced) demonstrated a different pattern: while maintaining a similar capillary density distribution to the other two groups, it showed distinct characteristics in flow velocity and myocardial blood flow between the septum and free wall. Specifically, the free wall exhibited increased flow velocity without significant differences in myocardial blood flow compared to the septum. This observation aligns with the previously mentioned multifactorial regulation of myocardial blood flow in pathological hypertrophy, where structural microcirculatory abnormalities, hemodynamic alterations, and myocardial metabolic products collectively influence both microcirculatory flow velocity regulation and blood flow distribution patterns [23, 24, 25].

However, it should be noted that some patients in the pathological hypertrophy group of this study received relevant pharmacological treatment, which may have potentially influenced the observed myocardial perfusion parameters. Current evidence suggests that myocardial microcirculatory dysfunction in hypertensive patients constitutes an important pathological basis for cardiovascular adverse events. Vasodilators and beta‐blockers, as antihypertensive agents, have been demonstrated to partially reverse the microcirculatory impairment associated with pathological myocardial hypertrophy [26, 27]. Additionally, effective blood pressure control contributes to reducing cardiac load and, to some extent, improves myocardial microcirculatory perfusion [28]. Consequently, the myocardial microcirculatory perfusion levels observed in this study may reflect the combined effects of pharmacological intervention and blood pressure management.

ROC curve analysis identified the A value as the most robust discriminator (AUC = 0.904) between hypertrophy types. With a cutoff > 8.13 dB providing 92.7% sensitivity, this parameter outperformed β and MBF in diagnostic accuracy. This supports the clinical utility of MCE for differentiating adaptive versus maladaptive hypertrophy before overt functional decline occurs.

### 6.1 Study Limitations

This investigation has several methodological constraints that warrant consideration: First, as a single‐center study with a relatively small sample size, the statistical power may be limited and the findings require validation in larger cohorts. Second, the pathological hypertrophy group in this study exclusively included patients with myocardial hypertrophy secondary to primary hypertension, without incorporating other etiologies such as hypertrophic cardiomyopathy or valvular heart disease. Given the focus on a single etiological spectrum, the generalizability of the findings is limited and may not apply to all types of pathological myocardial hypertrophy. Furthermore, it should also be noted that while clinically manifest HCM was systematically excluded in the physiological hypertrophy and control groups through comprehensive symptom evaluation, detailed medical history review, electrocardiographic analysis, and echocardiographic assessment, the study did not routinely employ advanced diagnostic modalities such as CMR or genetic testing. Consequently, the possibility of including individuals with early‑stage or subclinical HCM—particularly those who are genotype‑positive but phenotype‑negative—cannot be entirely ruled out. Given that such undetected cases may influence the study outcomes, especially within the athlete cohort, this limitation is explicitly acknowledged herein. Additionally, as this study was designed as an initial comparative analysis of myocardial perfusion between physiological and pathological hypertrophy groups, detailed data regarding antihypertensive medication types, treatment duration, and compliance were not systematically recorded. These factors may introduce confounding effects in the interpretation of microcirculatory parameters. Nevertheless, real‐time blood pressure measurements obtained during imaging confirmed that the pathological hypertrophy group exhibited significantly higher blood pressure levels compared to both the physiological hypertrophy group and the controls. Thus, the results presented here retain relevant insights into the microcirculatory profile associated with hypertensive myocardial hypertrophy. Future work will include dedicated studies on pathological hypertrophy to systematically examine how specific pharmacotherapeutic strategies modulate myocardial perfusion. Finally, the current understanding of the mechanisms regulating myocardial blood flow velocity in pathological hypertrophy remains incomplete, particularly regarding microcirculatory dysfunction and compensatory hemodynamic adaptations. Future research should integrate advanced imaging techniques, molecular biomarkers, and genetic screening to further elucidate the pathophysiological mechanisms underlying different hypertrophy phenotypes and improve the accuracy of participant classification.

## 7 Conclusions

The core finding of this study is that MCE can quantitatively assess myocardial perfusion under different myocardial hypertrophy conditions and successfully reveals distinct microcirculatory patterns between physiological and pathological hypertrophy subtypes. The results show that structurally, physiological hypertrophy primarily presents as adaptive EH, while pathological hypertrophy is predominantly characterized by CH. Functionally, physiological hypertrophy (e.g., in long‐distance runners) enhances myocardial perfusion through microvascular proliferation, leading to well‐maintained or even improved microcirculatory function. In contrast, pathological hypertrophy (e.g., in hypertensive patients) is fundamentally characterized by significant CMD, manifesting as impaired perfusion accompanied by compensatory regulatory mechanisms. Notably, myocardial blood volume (the A‐value), as a surrogate marker of capillary density, serves as a reliable discriminator between these two hypertrophy phenotypes. In summary, this study highlights the critical role of MCE‐derived quantitative microcirculatory function parameters in the differential diagnosis of left ventricular hypertrophy.

## Author Contributions

Sun Pin conceived and designed the study. Liu Chunyao and Jiang Ruihan implemented the study protocol, performed data analysis and statistical processing. Liu Chunyao drafted the initial manuscript. Li Kai contributed to the formal re‐analysis of the key data during the revision stage, performed the visualization, and participated in the drafting of critical sections of the revised manuscript (Writing – Original Draft). Sun Pin and Lin Mingming critically revised the manuscript for important intellectual content. Wang Xiaofan, Lv Qifeng, Song Xiaoxia, Tian Yu, and Sun Juanjuan were responsible for clinical data acquisition. All authors reviewed and approved the final version of the manuscript for submission.

## Ethics Statement

The study protocol was approved by the Ethics Committee of the Affiliated Hospital of Qingdao University (Approval No.: QYFYEC2025‐28).

## Conflicts of Interest

The authors declare no conflicts of interest.

## Data Availability

The data that support the findings of this study are available from the corresponding author upon reasonable request.
